# Addition of β-galactosidase boosts the xyloglucan degradation capability of endoglucanase Cel9D from *Clostridium thermocellum*

**DOI:** 10.1186/s13068-018-1242-5

**Published:** 2018-09-04

**Authors:** Jonathan Herlet, Wolfgang H. Schwarz, Vladimir V. Zverlov, Wolfgang Liebl, Petra Kornberger

**Affiliations:** 1 Department of Microbiology, Technical University of Munich, Emil-Ramann-Str. 4, 85354 Freising-Weihenstephan, Germany; 2 Institute of Molecular Genetics, Russian Academy of Science, Kurchatov Sq. 2, Moscow, 123182 Russia

**Keywords:** Xyloglucan (XG), β-Galactosidase, Endoglucanase, Enzyme synergism, Tamarind kernel powder (TKP), Biomass degradation

## Abstract

**Background:**

Increasing the efficiency of enzymatic biomass degradation is crucial for a more economically feasible conversion of abundantly available plant feedstock. Synergistic effects between the enzymes deployed in the hydrolysis of various hemicelluloses have been demonstrated, which can reduce process costs by lowering the amount of enzyme required for the reaction. Xyloglucan is the only major hemicellulose for which no such effects have been described yet.

**Results:**

We report the beneficial combination of two enzymes for the degradation of the hemicellulose xyloglucan. The addition of β-galactosidase Bga2B from *Clostridium stercorarium* to an in vitro hydrolysis reaction of a model xyloglucan substrate increased the enzymatic efficiency of endoglucanase Cel9D from *Clostridium thermocellum* to up to 22-fold. Furthermore, the total amount of enzyme required for high hydrolysis yields was lowered by nearly 80%. Increased yields were also observed when using a natural complex substrate—tamarind kernel powder.

**Conclusion:**

The findings of this study may improve the valorization of feedstocks containing high-xyloglucan amounts. The combination of the endoglucanase Cel9D and the β-galactosidase Bga2B can be used to efficiently produce the heptasaccharide XXXG. The exploitation of one specific oligosaccharide may open up possibilities for the use as a prebiotic or platform chemical in additional reactions.

**Electronic supplementary material:**

The online version of this article (10.1186/s13068-018-1242-5) contains supplementary material, which is available to authorized users.

## Background

The use of renewable plant biomass is a key to a viable bio-based economy. The efficient enzymatic hydrolysis of these bulk substrates into value-added products is a promising approach to achieve this goal. However, because lignocellulolytic polysaccharides show a complex and diverse composition, multiple enzyme classes are required for their hydrolysis [1, 2]. Furthermore, owing to the high price of enzymes, reducing their amount required in reactions is a major task in making bioconversions more cost-effective [3–6].

Synergism between endo- and exoglucanases has been described to lower the enzyme concentration required for cellulose degradation [6–9]. The addition of non-cellulolytic enzymes, such as xylanases, can further enhance the hydrolytic potential of cellulase mixtures [10–12] and increase the hydrolytic efficiency of pretreated bagasse by enhancing cellulose accessibility [13]. Similarly, the degradation of xylans, particularly arabinoxylans, can be improved by exploiting the synergistic effects of β-xylanases, β-xylosidases, and α-arabinofuranosidases [14–16]. Moreover, synergistic enzyme combinations have been described for the degradation of other hemicellulosic substrates, such as (galacto)mannan [17–19] and glucans [20]. However, while synergism between various enzyme activities has been described for the hydrolysis of all major hemicelluloses (xylans, glucans, and mannans), it has not yet been reported for xyloglucan (XG) breakdown.

Xyloglucan is a common polysaccharidic structural component predominant in the primary cell walls of plants [21, 22]. It comprises a β-1,4-glucan backbone that is regularly decorated with other sugar residues. XG interacts with cellulose and modulates cell wall rigidity; however, the extent of its tethering effect remains only partly understood [23]. In contrast, XG also serves as a storage polysaccharide in tamarind seeds and is usually hydrolyzed into four backbone oligosaccharides [24]: XXXG, XLXG, XXLG, and XLLG (these four-letter abbreviations represent hepta-, octa- and nonameric oligosaccharides with a Glc_4_ backbone and xylose and galactose substituents; for nomenclature code, see [6]). XG polysaccharides have various industrial and pharmaceutical applications, but the potential of XG oligosaccharides has not been intensively investigated to date although they have been shown to exhibit lipid-lowering properties in rats [25]. Thus, more effects in analogy to other oligosaccharides can be expected.

Endoglucanase Cel9D (EC 3.2.1.4) is active toward tamarind XG from *C. thermocellum* and rapidly liberates the hepta- and octasaccharides, XXXG, XLXG, and XXLG. In contrast, the nonasaccharide XLLG, which comprises two galactose residues, is released much more slowly. Other endoglucanases from *C. thermocellum* with activity toward XG did not show a difference in the release rate of the oligosaccharides. We presumed that Cel9D is hindered by the consecutive galactose substitutions. In this study, we therefore, investigated whether the addition of a β-galactosidase can facilitate a faster and complete XG degradation by Cel9D.

## Methods

### Protein production and specific activity

All enzymes used in this study were produced using the pET-24c(+) expression plasmid (Thermo Fisher Scientific, Waltham, USA) in *E. coli* BL21Star™ cells (Thermo Fisher Scientific) and purified by means of a C-terminal hexahistidine-tag as previously described [9] except for the use of auto-induction medium [26] instead of IPTG induction. The specific activity of Cel9D for XG (480 ± 20 U/mg) was determined as previously described using the DNSA-assay [27]. β-Galactosidase Bga2B (Cst_c09830, EC 3.2.1.23) from *C. stercorarium* which showed a specific activity of 3.1 U/mg for XG, was characterized and kindly supplied by Jannis Broeker of TU Munich, Germany.

### Enzyme reaction

The total reaction volume of 1.2 ml comprised 1 ml 1% (w/v) tamarind XG substrate obtained from Megazyme (Bray, Ireland) solubilized in ddH_2_O, 100 µl citrate–phosphate buffer pH 6.4, prepared as described previously [28], and 100 µl of the respective diluted enzymes (Cel9D, 0.12–24 U; Bga2B, 0–155 mU). All components were mixed on ice before starting the assay reaction at 60 °C. Suitable reaction conditions for both enzymes were determined using pH vs. temperature plots as described previously [28]. Incubation was performed by shaking at 800 rpm at 60 °C for 6 h using a HLC MHR 23 thermomixer (Ditabis, Pforzheim, Germany). After the first 5 min of the overall incubation time, the reaction tubes were inverted several times to ensure that the reaction samples were thoroughly mixed as the viscosity is reduced at the beginning of the reaction. Finally, the reaction tubes were cooled in ice water before they were either directly used for high-performance anion-exchange chromatography coupled with pulsed amperometric detection (HPAEC–PAD) analysis or frozen at − 20 °C. All reactions were performed in triplicate. Tamarind kernel powder (TKP) was obtained from Tamarind Magic (Hyderabad, India) and used as described for the model XG substrate.

### HPAEC–PAD analysis

Reaction yields of the enzyme reactions described above were assessed by quantifying the amount of Glc_4_ backbone oligosaccharides produced using HPAEC–PAD with a Dionex™ ICS-3000 system (Thermo Fisher Scientific). The reaction products obtained from the enzyme reactions with Cel9D and Bga2B contained all four Glc_4_ backbone oligosaccharides present in tamarind XG (XXXG, XLXG, XXLG, and XLLG) in varying ratios. These Glc_4_ backbone oligosaccharides are the end products of the reaction. All samples were heat treated for 30 min at 95 °C to completely inactivate Cel9D. To ensure an unbiased quantification, 100 µl aliquots of all samples were then incubated with 23 mU Bga2B at 60 °C for 6 h. This step ensures that the XLXG, XXLG, and XLLG oligosaccharides present in the reaction mixture are converted into the XXXG oligosaccharide. This procedure does not change the total amount of Glc_4_ backbone oligosaccharides produced and allows quantification via HPAEC–PAD against a XXXG oligosaccharide standard obtained from Megazyme.

5 µl of the aliquot was mixed with 495 µl ddH_2_O and analyzed by HPAEC–PAD on a CarboPac PA1 column using conditions previously described [29]. XG heptasaccharide XXXG was used as a standard to quantify the samples and as a bracketing standard to account for the loss of detector signal over time. Negative controls of the substrate without Cel9D were tested to demonstrate that no oligosaccharides were present or formed without the addition of the endoglucanase.

## Results and discussion

**Fig. 1:**
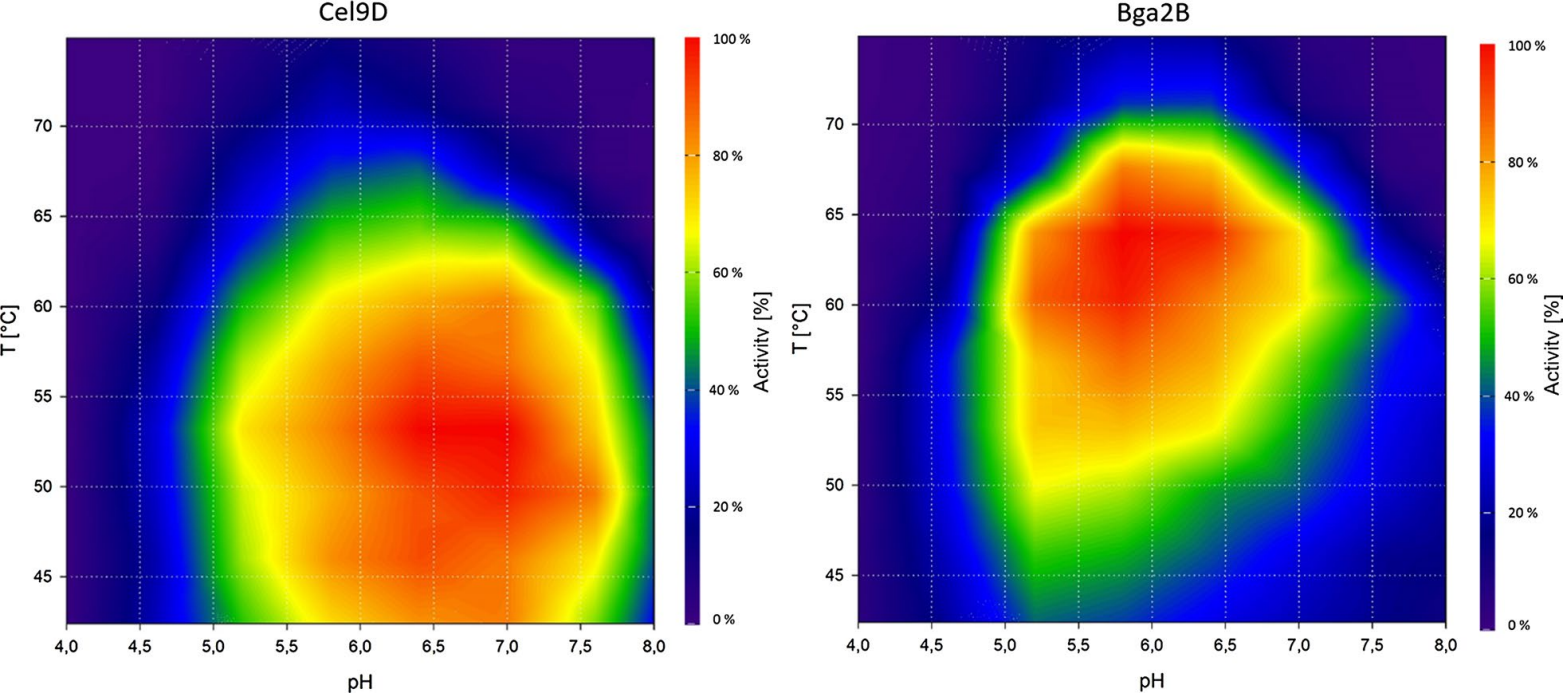
pH vs. temperature plots of Cel9D (left) and Bga2B (right) using XG and *p*NP-β-d-galactoside as substrate, respectively. The following gradient was used in the PCR cycler: 42.4, 46.0, 49.6, 53.2, 56.8, 60.4, 64.0, 67.6, 71.2, and 74.9 °C. The relative activity of the enzyme is depicted by color from red (100%) to purple (0%)

Glc_4_ backbone oligosaccharides are the end products of the reaction of XG with Cel9D. However, the enzyme produced XLLG more slowly than the other Glc_4_-backbone oligosaccharides (Additional file 1: Figure S1). We tested, whether the addition of the β-galactosidase Bga2B can increase the amount of Glc_4_ backbone oligosaccharides released by Cel9D. To ensure, that incubation conditions are applicable for both enzymes, we used a pH vs temperature activity profile (Fig. 1). Conditions suitable for both enzymes were visually determined to be pH 6.4 and 60 °C. To study the beneficial interaction between the two enzymes for XG hydrolysis, we first incubated a fixed amount of Cel9D (480 mU) with varying amounts of Bga2B (0–155 mU). With 31 mU Bga2B determined as the required amount, we varied the Cel9D concentration from 120 mU to 24 U to achieve a maximal reduction of Cel9D needed and achieved an 80% reduction in total enzyme load.

**Fig. 2:**
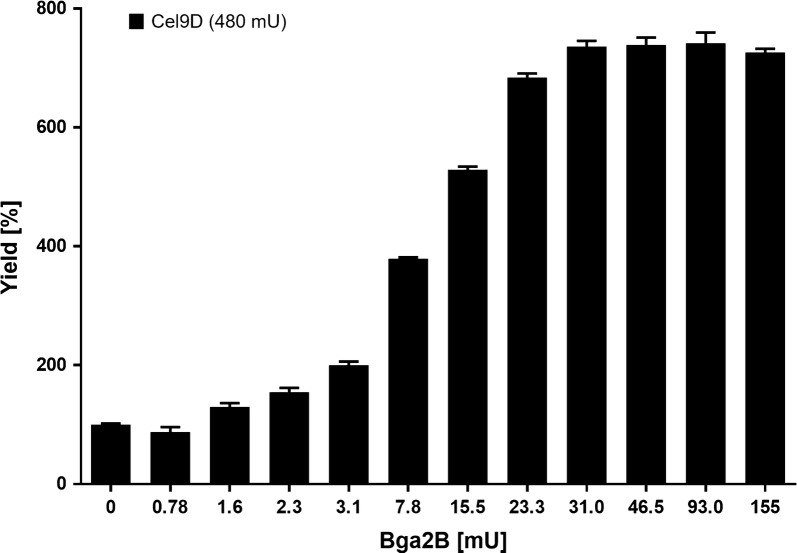
Relative yields of Glc_4_ XG oligosaccharides liberated from xyloglucan at increasing concentrations of β-galactosidase Bga2B. Reactions were carried out with 1% (w/v) substrate in a reaction volume of 1.2 ml at pH 6.4 and 60 °C. A constant amount (480 mU) of Cel9D was used in all reactions. The yield of the reaction without the addition of β-galactosidase Bga2B was set to 100%

To test whether the addition of a β-galactosidase improves the XG degradation capability of Cel9D, 480 mU of Cel9D were incubated with increasing amounts of Bga2B (0–155 mU). As shown in Fig. 2, the reaction yield during the incubation interval doubled with the addition of only 3.1 mU Bga2B and increased further to sevenfold when 31 mU or more Bga2B was added. The smallest amount of Bga2B to produce even a slight increase in reaction yield was determined to be 1.55 mU. Negative controls using only Bga2B without Cel9D showed the release of galactose and no degradation of the polymeric XG backbone (data not shown).

**Fig. 3:**
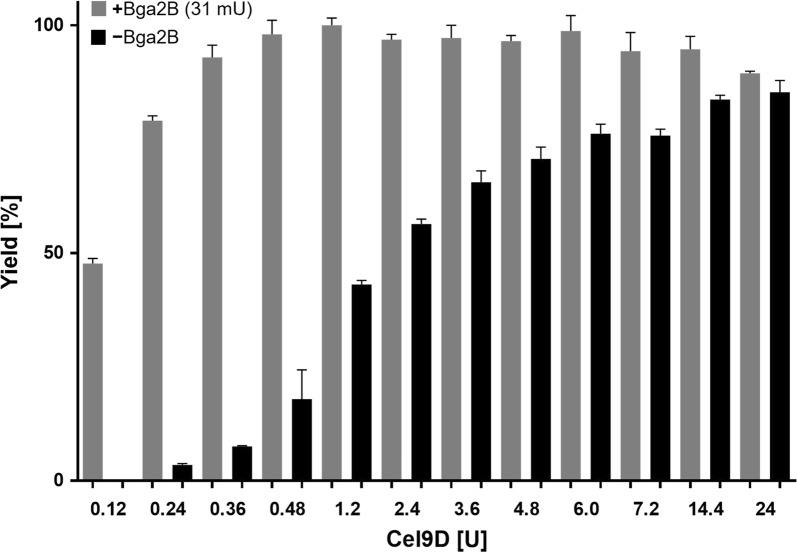
Relative yields of Glc_4_ XG oligosaccharides liberated from xyloglucan using increasing concentrations of the xyloglucanase Cel9D. Reactions were performed with either the addition of 31 mU β-galactosidase Bga2B (gray) or in its absence (black). The highest obtained reaction yield was set to 100%

In the next experiment, between 120 mU and 24 U of Cel9D were added to the reaction mix, with and without a constant addition of 31 mU of Bga2B (Fig. 3, gray bars and black bars respectively). In the absence of Bga2B, reaction yield correlated directly with Cel9D concentration. In contrast, in the presence of 31 mU Bga2B reaction yield reached nearly 100% using only 360 mU Cel9D. Therefore, the yield ratios between reactions with and without Bga2B were more pronounced at lower Cel9D concentrations, peaking at more than a 22-fold yield increase for the reaction containing 240 mU Cel9D.

Interestingly, even with the highest amount of Cel9D used in this study the XG backbone cleavage reaction could not be completed to the extent achievable with Bga2B addition. Using 24 U of only Cel9D (50 µg) resulted in an 85.3 ± 2.6% reaction yield. In contrast, only 360 mU of Cel9D (0.75 µg) produced a yield of 93 ± 2.7% when combined with 31 mU Bga2B (10 µg). Thus, in addition to the increased yield, the total amount of enzyme in the reaction was concomitantly reduced by nearly 80%. This may indicate that Cel9D is unable to efficiently degrade the parts of the XG substrate that contain a high abundance of double galactose substitutions (XLLG). This negative effect is alleviated by removing the side chains with the β-galactosidase. Bga2B was the only enzyme with β-galactosidase activity toward XG available in our lab. Screening for a more active β-galactosidase could further lower the amount of enzyme needed in the reaction.

All other endo- and xyloglucanases from *C. thermocellum*, apart from Cel9D, with specific activities toward XG in a similar range (Xgh74A, Cel5E, and Cel9/44 J), were analyzed for a stimulating effect of galactosyl moiety removal, but none exhibited an increase in reaction yield from the addition of Bga2B. With the addition of 31 mU Bga2B, Cel9D was the most effective XG degrading enzyme from *C. thermocellum* (Additional file 1: Figure S2). Additionally, an enzyme from *Herbivorax saccincola* GGR1^T^ with activity toward XG was tested, which revealed that Cel9K from this recently isolated bacterium [30] acts in a manner similar to Cel9D (unpublished data). Therefore, this effect is not limited to Cel9D from *C. thermocellum*, but may be specific to a certain subset of GH9 endoglucanases. Finding the underlying structural associations that produce this dependency would be an interesting task for future studies to further define this subset of enzymes.

The removal of the galactose moieties from the side chains of the XG substrate is a prerequisite for its complete hydrolysis to monosaccharides [31, 32], which makes the beneficial effects of synergism between the β-galactosidase Bga2B and endoglucanase Cel9D observed in this study particularly useful. With tamarind XG as a substrate, the reaction using these two enzymes in appropriate amounts for a complete reaction results in only two major reaction products: the heptasaccharide XXXG and the monosaccharide galactose. Galactose could easily be removed via various approaches, such as nanofiltration or simulated moving bed chromatography, leaving the XXXG oligosaccharide as the major product. The selective production of one large oligosaccharide with a defined degree of polymerization implies the potential for industrial application, including use as a platform chemical. Additional properties, such as application as prebiotic food or feed supplement, should be evaluated. The addition of Bga2B increased the total product yield obtained using the model XG substrate as well as tamarind kernel powder (TKP, Additional file 1: Figure S3). TKP is a bulk substrate and a side product of the tamarind pulp industry and may serve as a cheap substrate for industrial applications.

In conclusion, our study demonstrates the first beneficial combination of two enzymes for the degradation of the hemicellulose XG. β-Galactosidase Bga2B addition during the endoglucanase Cel9D-catalyzed hydrolysis of XG boosted the reaction yield and lowered the total amount of enzyme needed. The effect described in this study could be applied in the valorization of substrates with high XG contents, such as TKP.

## Additional file

**Additional file 1: Figure S1.** Relative peak areas determined by HPAEC-PAD of the XG oligosaccharides XXXG, XLXG/XXLG, and XLLG over time during XG hydrolysis using Cel9D. **Figure S2.** Relative reaction yields determined by HPAEC-PAD with 1 µg Cel9D, Cel5E, Cel9/44J or Xgh74A using standard reaction conditions with (grey) and without (black) the addition of 31 mU Bga2B. **Figure S3.** XXXG oligo concentrations determined by HPAEC-PAD after 6 h, 60 °C incubation of 480 mU Cel9D with (grey) and without (black) the addition of 31 mU Bga2B.

## References

[1] Percival Zhang YH, Himmel ME, Mielenz JR (2006). Outlook for cellulase improvement: screening and selection strategies. Biotechnol Adv.

[2] Koeck DE, Pechtl A, Zverlov VV, Schwarz WH (2014). Genomics of cellulolytic bacteria. Curr Opin Biotechnol.

[3] Khare SK, Pandey A, Larroche C (2015). Current perspectives in enzymatic saccharification of lignocellulosic biomass. Biochem Eng J.

[4] Antonov E, Schlembach I, Regestein L (2017). Process relevant screening of cellulolytic organisms for consolidated bioprocessing. Biotechnol Biofuels.

[5] Klein-Marcuschamer D, Oleskowicz-Popiel P, Simmons BA, Blanch HW (2012). The challenge of enzyme cost in the production of lignocellulosic biofuels. Biotechnol Bioeng.

[6] Viikari L, Alapuranen M, Puranen T (2007). Thermostable enzymes in lignocellulose hydrolysis. Adv Biochem Eng Biotechnol.

[7] Kostylev M, Wilson D (2012). Synergistic interactions in cellulose hydrolysis. Biofuels.

[8] Van Dyk JS, Pletschke BI (2012). A review of lignocellulose bioconversion using enzymatic hydrolysis and synergistic cooperation between enzymes-Factors affecting enzymes, conversion and synergy. Biotechnol Adv.

[9] Leis B, Held C, Bergkemper F (2017). Comparative characterization of all cellulosomal cellulases from *Clostridium thermocellum* reveals high diversity in endoglucanase product formation essential for complex activity. Biotechnol Biofuels.

[10] Hu J, Arantes V, Pribowo A, Saddler JN (2013). The synergistic action of accessory enzymes enhances the hydrolytic potential of a “cellulase mixture” but is highly substrate specific. Biotechnol Biofuels.

[11] Hu J, Arantes V, Saddler JN (2011). The enhancement of enzymatic hydrolysis of lignocellulosic substrates by the addition of accessory enzymes such as xylanase: is it an additive or synergistic effect?. Biotechnol Biofuels.

[12] Zhang J, Viikari L (2014). Impact of xylan on synergistic effects of xylanases and cellulases in enzymatic hydrolysis of lignocelluloses. Appl Biochem Biotechnol.

[13] Gonçalves GAL, Takasugi Y, Jia L (2015). Synergistic effect and application of xylanases as accessory enzymes to enhance the hydrolysis of pretreated bagasse. Enzyme Microb Technol.

[14] Yang X, Shi P, Huang H (2014). Two xylose-tolerant GH43 bifunctional β-xylosidase/α-arabinosidases and one GH11 xylanase from *Humicola insolens* and their synergy in the degradation of xylan. Food Chem.

[15] Kang DH, You SK, Joo YC (2018). Synergistic effect of the enzyme complexes comprising agarase, carrageenase and neoagarobiose hydrolase on degradation of the red algae. Bioresour Technol.

[16] Huang D, Liu J, Qi Y (2017). Synergistic hydrolysis of xylan using novel xylanases, β-xylosidases, and an α-l-arabinofuranosidase from *Geobacillus thermodenitrificans* NG80-2. Appl Microbiol Biotechnol.

[17] Jeon SD, Yu KO, Kim SW, Han SO (2011). A celluloytic complex from *Clostridium cellulovorans* consisting of mannanase B and endoglucanase E has synergistic effects on galactomannan degradation. Appl Microbiol Biotechnol.

[18] Malgas S, van Dyk JS, Pletschke BI (2015). A review of the enzymatic hydrolysis of mannans and synergistic interactions between β-mannanase, β-mannosidase and α-galactosidase. World J Microbiol Biotechnol.

[19] Malgas S, van Dyk SJ, Pletschke BI (2015). β-Mannanase (Man26A) and α-galactosidase (Aga27A) synergism—a key factor for the hydrolysis of galactomannan substrates. Enzyme Microb Technol.

[20] Driskill LE, Bauer MW, Kelly RM (1999). Synergistic interactions among β-laminarinase, β-1,4-glucanase, and β-glucosidase from the hyperthermophilic archaeon *Pyrococcus furiosus* during hydrolysis of β-1,4-, β-1,3-, and mixed-linked polysaccharides. Biotechnol Bioeng.

[21] Cosgrove DJ (2005). Growth of the plant cell wall. Nat Rev Mol Cell Biol.

[22] Eklöf JM, Ruda MC, Brumer H (2012). Distinguishing xyloglucanase activity in endo-β(1 → 4)glucanases. Methods Enzymol.

[23] Park YB, Cosgrove DJ (2015). Xyloglucan and its interactions with other components of the growing cell wall. Plant Cell Physiol.

[24] York WS, Harvey LK, Guillen R (1993). Structural analysis of tamarind seed xyloglucan oligosaccharides using β-galactosidase digestion and spectroscopic methods. Carbohydr Res.

[25] Yamatoya K, Shirakawa M, Kuwano K (1996). Effects of hydrolyzed xyloglucan on lipid metabolism in rats. Food Hydrocoll.

[26] Studier FW (2005). Protein production by auto-induction in high density shaking cultures. Protein Expr Purif.

[27] Mechelke M, Koeck DE, Broeker J (2017). Characterization of the arabinoxylan-degrading machinery of the thermophilic bacterium *Herbinix hemicellulosilytica*—six new xylanases, three arabinofuranosidases and one xylosidase. J Biotechnol.

[28] Herlet J, Kornberger P, Roessler B (2017). A new method to evaluate temperature vs. pH activity profiles for biotechnological relevant enzymes. Biotechnol Biofuels.

[29] Mechelke M, Herlet J, Benz JP (2017). HPAEC-PAD for oligosaccharide analysis—novel insights into analyte sensitivity and response stability. Anal Bioanal Chem.

[30] Koeck DE, Mechelke M, Zverlov VV (2016). Herbivorax saccincola gen. nov., sp. nov., a cellulolytic, anaerobic, thermophilic bacterium isolated via *in sacco* enrichments from a lab scale biogas reactor. Int J Syst Evol Microbiol.

[31] De Alcântara PHN, Dietrich SMC, Buckeridge MS (1999). Xyloglucan mobilisation and purification of a (XLLG/XLXG) specific β-galactosidase from cotyledons of *Copaifera langsdorffii*. Plant Physiol Biochem.

[32] Ravachol J, De Philip P, Borne R (2016). Mechanisms involved in xyloglucan catabolism by the cellulosome-producing bacterium *Ruminiclostridium cellulolyticum*. Sci Rep.

