# Supplementary material for: Addition of β-galactosidase boosts the xyloglucan degradation capability of endoglucanase Cel9D from Clostridium thermocellum
Source: Biotechnol Biofuels. 2018 Sep 4;11:238. doi: 10.1186/s13068-018-1242-5 (PMC6122707; doi:10.1186/s13068-018-1242-5)
Supplement: Supplementary file 1 — Additional file 1: Figure S1. Relative peak areas determined by HPAEC-PAD of the XG oligosaccharides XXXG, XLXG/XXLG, and XLLG over time during XG hydrolysis using Cel9D. Figure S2. Relative reaction yields determined by HPAEC-PAD with 1 µg Cel9D, Cel5E, Cel9/44J or Xgh74A using standard reaction conditions with (grey) and without (black) the addition of 31 mU Bga2B. Figure S3. XXXG oligo concentrations determined by HPAEC-PAD after 6 h, 60 °C incubation of 480 mU Cel9D with (grey) and without (black) the addition of 31 mU Bga2B. [file 13068_2018_1242_MOESM1_ESM.docx]

**Figure S1** Relative peak areas determined by HPAEC-PAD of the xyloglucan oligosaccharides XXXG, XLXG+XXLG, and XLLG over time during xyloglucan hydrolysis using Cel9D.


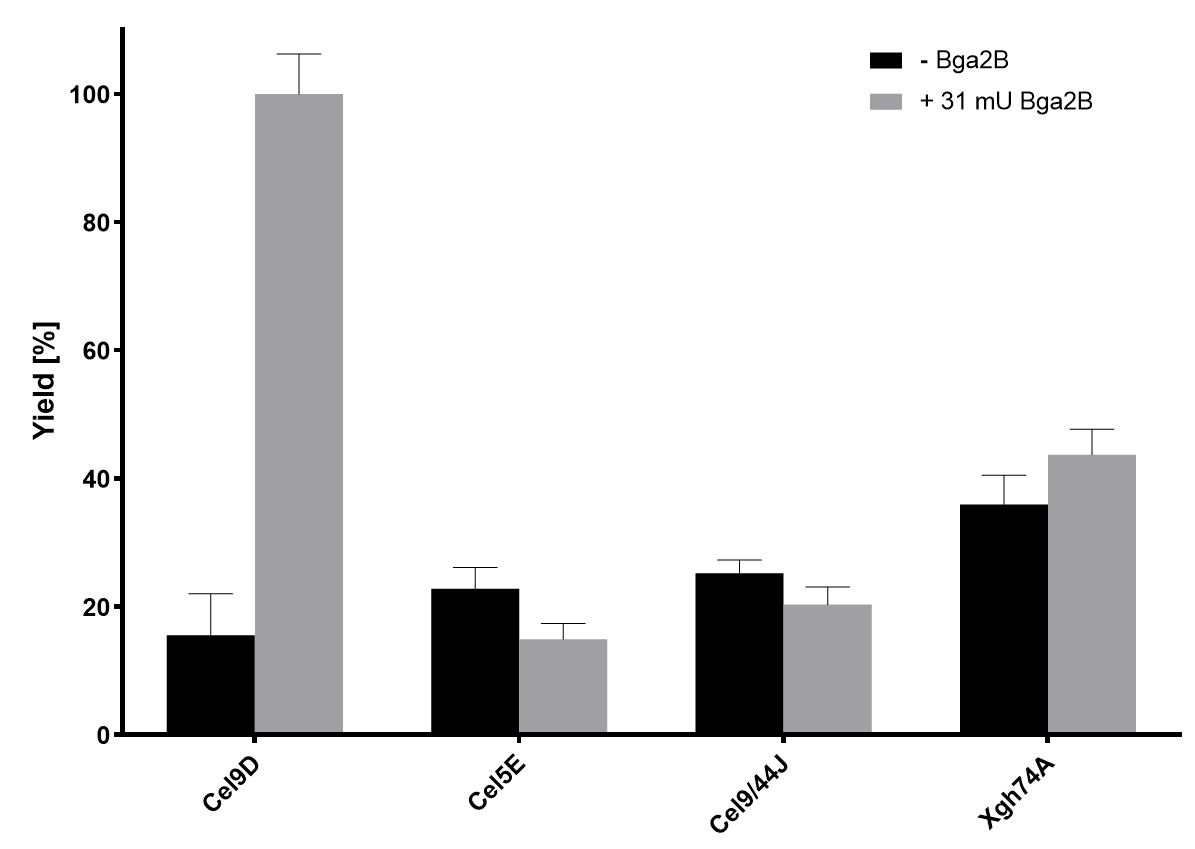


**Figure S2** Relative reaction yields determined by HPAEC-PAD with 1 µg Cel9D, Cel5E, Cel9/44J or Xgh74A using standard reaction conditions with (grey) and without (black) the addition of 31 mU Bga2B.

**Figure S3** XXXG concentrations determined by HPAEC-PAD after 6h, 60°C incubation of 480 mU Cel9D with (grey) and without (black) the addition of 31 mU Bga2B.
